# The Need for a Specific Risk Prediction System in Native Valve Infective Endocarditis Surgery

**DOI:** 10.1100/2012/307571

**Published:** 2012-03-12

**Authors:** Marisa De Feo, Maurizio Cotrufo, Antonio Carozza, Luca S. De Santo, Francesco Amendolara, Salvatore Giordano, Ester E. Della Ratta, Gianantonio Nappi, Alessandro Della Corte

**Affiliations:** ^1^Department of Cardiothoracic Sciences, Second University of Naples, c/o V Monaldi Hospital, Via L. Bianchi, 80131 Naples, Italy; ^2^Department of Cardiovascular Surgery, “Pineta Grande” Hospital, 81030 Castel Volturno, Italy

## Abstract

The need for a specific risk score system for infective endocarditis (IE) surgery has been previously claimed. In a single-center pilot study, preliminary to future multicentric development and validation, bivariate and multivariate (logistic regression) analysis of early postoperative mortality predictors in 440 native valve IE patients were performed. Mathematical procedures assigned scores to the independent predictors emerged (AUC of the ROC curve: 0.88). Overall mortality was 9.1%. Six predictors were identified and assigned scores, including age (5–13 points), renal failure (5), NYHA class IV (9), critical preoperative state (11), lack of preoperative attainment of blood culture negativity (5), perivalvular involvement (5). Four risk classes were drawn ranging from “very low risk” (≤5 points, mean predicted mortality 1%), and to “very high risk” (≥20 points, 43% mortality). IE-specific risk stratification models are both needed, as disease-specific factors (e.g., cultures, abscess), beside the generic ones (e.g., age, renal impairment) affect mortality, and feasible.

## 1. Introduction

Surgical prognosis of infective endocarditis (IE) is heterogeneous, in as much as different microbiologic etiologies; baseline conditions and modes of presentation to surgery can combine variably to give rise to disparate patient profiles with different risk of postoperative mortality. Likely owing to this heterogeneity, a number of retrospective studies have yielded discordant results in terms of impact and timing of surgery [1–3].

In this scenario, prognostic stratification tools could be helpful both in case-by-case clinical practice, to assist in the decision on surgical indication, and in clinical research, to warrant homogeneity of cohorts in comparative studies and to identify unique patient profiles in whom to assess surgical outcomes [1–3]. A specific prognostic tool for IE is not currently in use; thus, perioperative risk prediction for IE represents an area still open to further improvement [4, 5]. Different scoring systems are used in cardiac surgery for prognostic classification, including the additive and logistic models of the European System for Cardiac Operative Risk Evaluation (EuroSCORE) [6], the Society of Thoracic Surgeons (STS) scores [7], and the New York models [8]. However, none of them is specific for the setting of endocarditis, although surgery for endocarditis is not comparable to other cardiac procedures in terms of postoperative morbidity and mortality risks [5]. The first large study addressing the need for a dedicated stratification method has been very recently issued by the Duke University group, who substantially performed a recalibration of the STS system in a selected population with IE [9]. 

One aim of the present study was to verify whether disease-specific determinants of mortality risk following native valve IE surgery could be detected from a review of our experience. Another aim was to assess the methodological feasibility of developing a specific risk score system for native valve IE, as a preliminary study, foreseeing future application of the method in a more statistically powered multicentric study.

## 2. Materials and Methods

### 2.1. Patient Series and Variables

A dedicated database was prospectively filled in from January 1980 through December 2009 to save the data concerning 591 operations for infective endocarditis performed at our tertiary referral Institution. For sake of sample homogeneity with respect to preoperative conditions and risk of postoperative mortality [10], only cases of IE of native valves were considered for the present analysis, excluding 151 cases of prosthetic IE. Thus, 440 patients constituted the study population (mean age 49 ± 16 years, range 8–87, 72% males).

Preoperative variables included in the database concerned activity phase (active/healed endocarditis, where “active” is defined as endocarditis still under antibiotic therapy or with positive blood cultures or fever, leukocytosis, raised inflammation markers); valve (or valves) involved; type of anatomic lesion (vegetation, cusp perforation, or perivalvular involvement, including abscesses and fistulae); risk factors for endocarditis development (drug abuse, immunodeficiency, congenital or acquired valve anomalies, dialysis); comorbidities (renal failure or serum creatinine >2 mg/dL at admission, chronic liver disease, diabetes, hypertension, chronic pulmonary disease); basic echocardiography parameters (left ventricle dimensions, septum thickness, ejection fraction); clinical presentation (including New York Heart Association (NYHA) functional class, previous embolisms, septic/cardiogenic shock, preoperative ventilatory support, either invasive or noninvasive); surgical indication criteria (elective, urgent, or on emergency). From January 2000 (when the EuroSCORE was implemented in the Institutional protocols) to the end of the data collection period, the logistic and additive EuroSCORE values had been recorded for each patient as well, along with the relative criteria. The following microbiology variables from the database were used for the present analysis: preoperative blood cultures categorized as “ever positive” or “always negative” (data available for 95% patients); positivity/negativity of the latest blood culture before operation; microbial species isolated from blood. Included in the database but not in the predicting models for mortality were positivity/negativity of valve tissue cultures (data available for 97.7% patients) and microbial species isolated postoperatively from the surgical specimens.

Surgical methods remained substantially the same throughout the study period. Standard cardiopulmonary bypass methods were always employed, protecting the heart by means of cold crystalloid cardioplegia in all cases. 

Among the recorded postoperative outcomes, postoperative mortality (defined as 30-day death or in-hospital death, if the patient had a hospital stay longer than 30 days before dying), cause of death, postoperative evidence of non-eradication (positive blood cultures, fever, leukocytosis, inflammatory biomarkers including erythrosedimentation rate and C-reactive protein) were considered for the present study, whereas follow-up data were not used. To correct for possible time effects, a further variable was included in all analyses, indicating the decade of operation (1980–1989, 1990–1999, or 2000–2009). The present study was approved by the local Institutional Ethics Committee.

### 2.2. Statistical Methods for Score System Development

To assess the predictors of mortality and to experiment with the process of scoring system development, the above series was analyzed through a 3-step procedure, employing the SPSS ver13.0 statistical software (SPSS, Chicago, Ill, USA).

The first step consisted of the identification of the preoperative variables significantly associated, in bivariate analysis, with postoperative mortality. The *t*-test was used for comparison of continuous variables and the *χ* ^2^ test, or Fischer's exact test when appropriate, for categorical variables. Age was also categorized in 10-year classes. Significance was considered for a *P* value of at least 0.05. 

Variables resulting significantly associated with mortality were then entered (second step) in a multivariable logistic regression model predicting postoperative mortality. The Hosmer and Lemeshow test and the receiver operating characteristic (ROC) curve were used to estimate the fit and discrimination of the model. In this second step, the predicted probability of death of each patient was also yielded by the regression model and saved as a separate variable. 

According to a previously validated method [8, 11], the third step (risk index development) consisted of defining a constant, corresponding to the unitary risk in the score system: this was obtained by multiplying the coefficient estimate of the age variable by half the width of the age classes, that is, 0.041 × 5 years = 0.205. The coefficient for each identified risk factor was then divided by the unitary score (0.205) and rounded to the closest integer to be transformed into risk points (e.g., for the renal failure variable the coefficient was 1.076: 1.076/0.205 = 5.248 *≈* 5 points). The total risk score for each patient was then calculated as the sum of the points for each risk factor present in that patient. The correlation between total scores and predicted probability of death according to the regression model was assessed through Spearman's test, and it reached a *rho* value of 0.94 (*P* < 0.001).

For the subpopulation in which the EuroSCORE was prospectively calculated (252 patients operated on between 2000 and 2010), the correlation between the EuroSCORE and our system was evaluated by means of Kendall's *tau* correlation test (after preliminary assessment of nonnormality of distributions). The discriminating abilities of the two models were reevaluated in this subset by estimating the respective areas under the ROC curves.

## 3. Results

### 3.1. Baseline Features and Clinical Outcome

Endocarditis was in active phase in 365 (83%) patients, healed in 75. Endocarditis involved the native aortic valve alone in 200 (45.5%) cases, the mitral valve in 110 (25%), the tricuspid valve in 41 (9.3%), the pulmonary valve in 3 (0.7%), whereas multiple valve involvement was observed in the remaining 86 cases (the most frequent association being mitral plus aortic site, 71 patients, 16%). Among risk factors, diabetes was present in 51 patients (11.6%), intravenous drug abuse in 58 (13.2%), and renal failure (either acute or chronic) in 60 (13.6%). Surgery was indicated on an emergency basis, either for unresponsive septic state, severe cardiac failure, or severe systemic embolic risk, in 55 patients (12.5%), on urgency basis in 352 patients (80%), and only 33 patients were operated on electively.

Microorganisms identified in culture-positive cases (64.5%) included streptococcal spp. (43%), staphylococcal spp. (37%), gram negative spp. (14%), and others (6%, including, mainly, enterococci, fungi, corynebacteria, and polymicrobial etiologies).

Valve repair was performed in 48 patients (11%) in whom the valve tissue was judged not extensively involved by the infective process.

Overall postoperative mortality was 9.1% (40 patients). A cardiac cause of death (including low output syndrome, intractable arrhythmia, pulmonary embolism) was identified in 20 patients; other causes of mortality included multiorgan failure (9 patients), persistence of septic shock (6 patients), and postoperative pneumonia (5 patients).

### 3.2. Development of the Prognostic Classification System

**Figure 1:**
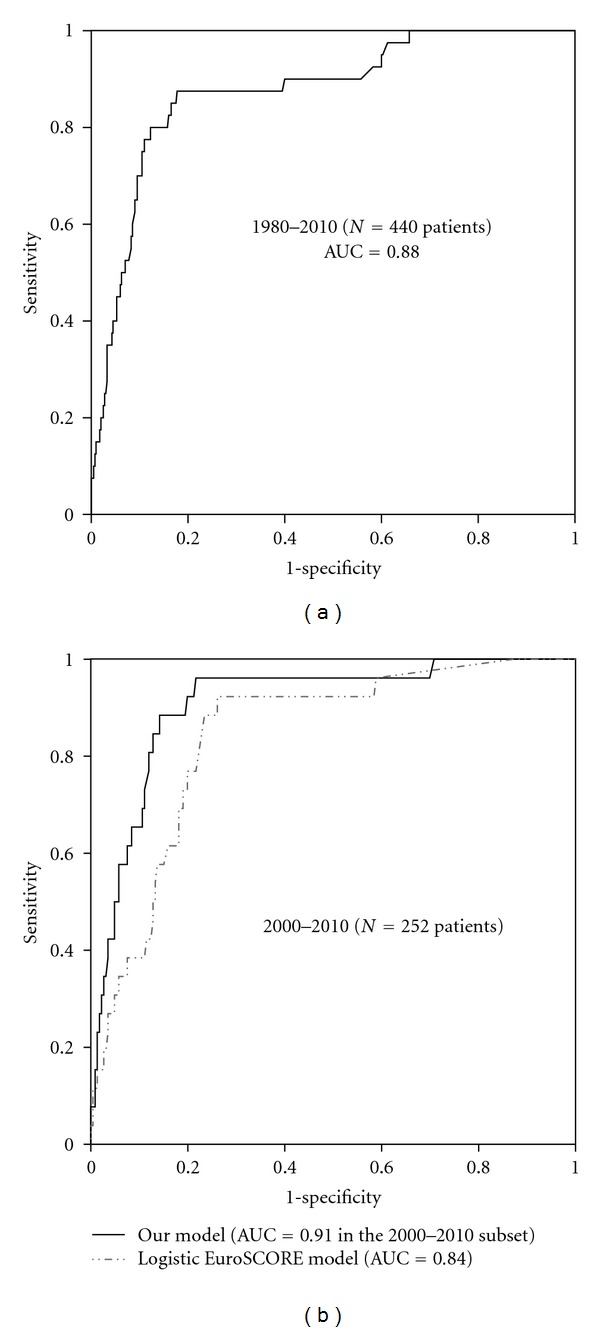
(a) Receiver-operator characteristic (ROC) curve for our logistic regression model developed from the analysis of 440 native valve IE patients: note the high value of the AUC, also compared to the one reported in the EuroSCORE development study (0.78 [8]); (b) ROC curves for our logistic regression model and the logistic EuroSCORE in the subpopulation (2000–2010) for whom EuroSCORE data were available; AUC: area under the curve.

Bivariate analysis results are displayed in Table 1. Only variables with *P* value <0.05 were introduced in the logistic regression model. The results of the multivariable analysis are presented in Table 2. Six independent predictors of mortality emerged in this series: age, preoperative renal failure, NYHA class IV, preoperative mechanical ventilatory assistance, perivalvular involvement (abscess or mycotic aneurysm) and positivity of the latest blood culture before operation. The *P* value for the Hosmer and Lemeshow test was 0.52, and the area under the ROC curve was 0.88 (95% CI 0.82–0.93), indicating satisfying goodness of fit and discriminating ability of the model (Figure 1(a)).

**Figure 2:**
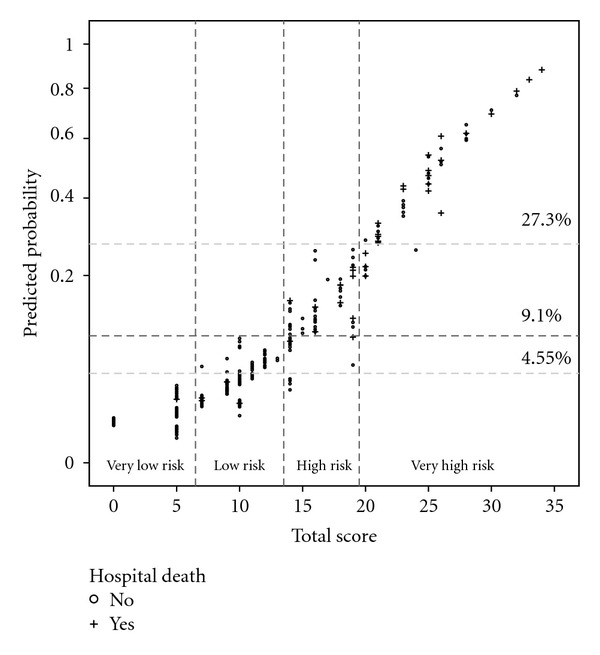
Scatterplot of predicted probability of postoperative 30-day death (*y*-axis, depicted with exponential scale) against total score per patient (*x*-axis). Horizontal dotted lines indicate the levels of death probability corresponding to the average observed mortality (9.1%), half of it (4.55%), and 3-fold higher mortality (27.3%). The four prognostic classes (thresholds indicated by vertical dotted lines) were identified by (1) the scores corresponding to a predicted mortality not exceeding 4.55% (0 or 5 points); (2) the score range including cases with a predicted mortality above 4.55%, but none beyond 9.1% (7–13 points); (3) the score range whereby predicted mortalities exceeded 9.1%; however, with no case over 27.3% (14–19 points); (4) the scores associated with predicted mortality range exceeding 27.3% (score ≥20).

Through the aforementioned method, scores were assigned to these variables, as depicted in Table 2. The scatterplot of the relation between predicted death probability and patients' total scores allowed for identification of risk classes: in particular, we referred to the upper limit of predicted mortality ranges with any given total score, following a criterion of clinical meaningfulness (Figure 2). Thus, the following prognostic groups were defined: total score = 0 or 5 (class 1, “very low risk”); 7–13 (class 2, “low risk”); 14–19 (class 3, “high risk”); ≥20 (class 4, “very high risk”). The four classes corresponded to the mean predicted mortality rates reported in Table 3 (*P* < 0.001 for all post-hoc comparisons with one-way analysis of variance and Bonferroni's correction). Observed mortality was 0.7% in class 1 (1/140 patients), 2.2% in class 2 (4/179 patients), 18% in class 3 (12/67 patients), and 42.6% in class 4 (23/54 patients) (*P* < 0.001). Comparisons between the four classes for clinically relevant variables (Table 3) showed that, beside the identified independent predictors, other variables resulted to significantly differ between risk classes, for example, diabetes and emergency. 

 In the subpopulation (*n* = 252) operated on from 2000 to 2010, correlations between the additive EuroSCORE and our score, as well as between logistic EuroSCORE and predicted probability derived from our logistic regression, were statistically significant, but the *tau* coefficients indicated that colinearity was not so high (0.47 both, *P* < 0.001). When the ROC curve analysis was repeated in this subgroup, it confirmed the good discrimination power of our model, with an AUC of 0.91 (95% CI 0.85–0.97), while the AUC was 0.84 (95% CI 0.77–0.91) for the logistic EuroSCORE in the same subgroup (Figure 1(b)).

## 4. Discussion

### 4.1. The Need for a Dedicated Score System

Methods of risk stratification in cardiac surgery serve to provide the patients with personalized prognosis prediction, to inform the clinical decision-making process, and to establish benchmarks for outcomes estimation and comparison. 

The present study, consistently with a number of previous analyses [12–15], showed that 2 out of the 6 most significant independent predictors of postoperative death in 440 endocarditis surgery patients were specific of the IE setting, that is, microbiology- or infection-related, and not included in the most commonly employed prognostic systems. 

Until recently, only a small Brazilian study including 186 patients had tried to derive a score system from a mixed native and prosthetic endocarditis cohort [12]: single-center design and small numbers prevent direct wider applicability of that system, as well as ours. The aforementioned Duke's study by Gaca and colleagues, conversely, was based on more than 13,600 IE patients from the STS database [9]: the multicentric design and the large sample size provided optimal statistical strength and applicability at least over all North America. However, STS entries do not include microbiological (cultures, species) and anatomical (vegetations, abscess) data, which; however, play an important prognostic role [12–15]. Moreover, the Duke study considered native and prosthetic valve IE together [9], whereas prosthetic valve endocarditis is a definitely distinct condition, with a considerably higher mortality and, even more importantly when prognostic stratification is concerned, with markedly different preoperative features [10]. Drawing a parallel, the widely employed EuroSCORE model for 30-day mortality prediction in cardiac surgery was first developed from a population of more than 19000 patients, predominantly undergoing coronary artery bypass grafting [6]. Its performance in peculiar subsets of patients, for example, in thoracic aorta surgery, has been demonstrated to be poorer, unless the system is modified to include additional disease-specific variables [16]. Similarly, IE-specific factors should probably be integrated into the score system developed by Gaca, or it could be combined with a complementary system accounting for them, to improve prediction (a C statistic of nearly 0.76 was reported [9]).

In the present study, including only native valve IE, we noticed that mortality in a predominant portion (risk classes 1 and 2, accounting together for 72%) of the whole series was not higher than that commonly observed following valve replacement for noninfectious disease (5 deaths, 1.6%). A distinct smaller patient group, representing the remaining 28% of our study population, showed a significantly higher mortality (approaching 29%), leading to an overall mortality of 9.1%. This suggests that native IE should not be considered as a high-risk condition in absolute terms; more appropriately, subsets of patients with clearly different prognosis, owing to differences in variables missing in the EuroSCORE or STS, should be otherwise distinguished. An even greater prognostic variability would have been found if also prosthetic endocarditis had been included. Notably, the EuroSCORE [6], STS score for valve surgery [7], and Duke's score for IE [9] only include one variable related to this specific setting, namely, the “active” endocarditis variable, whereas in the IE patient population, according to both our and others' studies [12–14], this does not result to independently predict mortality.

### 4.2. The Criteria in Our Score System

The factors included by our score system covered all three prognostically relevant aspects of patient presentation, that is, the baseline features, the degree of hemodynamic impairment, and the characteristics of the infectious process.

Among baseline conditions, age and renal failure were the only factors independently predicting mortality in this series. It is important to underscore the different categorization of the age variable between our score and the traditional systems: in the EuroSCORE a threshold of 60 years, in the STS of 55, marks the increase of risk compared to the younger ages; however, due to the peculiar epidemiology of native valve IE [2, 4, 12, 15], those higher thresholds may leave the prognostic differences between lower age groups unrecognized. 

The significance of renal failure as an independent predictor of mortality is not unique to the IE setting; nevertheless, it emerged in several previous reports on this topic [9, 13, 17] suggesting that its statistical weight as a prognostic factor may be even greater for IE operations than for general cardiac surgery. In fact, highest-risk patients often reach surgery after a more or less acute hemodynamic impairment, with possible renal hypoperfusion, and/or following a course of aggressive medical therapy, possibly including nephrotoxic antibiotics. Thus, renal failure may represent not only a predisposing factor for potentially lethal postoperative complications but also an important marker of high-risk status in IE. No other baseline conditions resulted to significantly predict mortality in our multivariable logistic regression; notably the analysis was also reperformed in the 2000–2010 subset adding the EuroSCORE value as a covariate (data not shown); however, the same 6 determinants resulted and the EuroSCORE did not reach significance as a predictor.

Among hemodynamics-related factors, only NYHA class IV, instead of other imaging (e.g., low ejection fraction) or clinical factors (e.g., preoperative inotropes), was a significant prognostic predictor of 30-day mortality. This result is consistent with the study by Hasbun and colleagues [4], proposing a risk classification method for medically or surgically treated complicated left-sided native IE: “congestive heart failure” was among the significant predictors in their scoring system, defined basically through physical findings, such as rales on examination and dyspnea at rest. 

Patients with preoperative ventilatory support in the present study were generally those with the most compromised hemodynamics: the indication to mechanical ventilation/support was pulmonary edema in 60% cases, septic shock, or both in the others. Notably, when this factor entered the multivariable logistic model, it suppressed the significance of the “emergency” factor, since all preoperatively intubated patients were operated on an emergency basis. Therefore, the importance of this factor in our system is consistent with what was found by the Duke study [9], whereby the preoperative hemodynamic condition of the patient was the greatest predictor of mortality.

With regard to microbiological and infection-related parameters, in our bivariate analysis, partly in accordance with the study by Hasbun and coworkers [4], an etiological agent different from *Streptococcus* and *Staphylococcus* was associated with higher mortality: this is also plausible according to clinical experience and published literature [18]. However, this factor did not emerge as an independent determinant in logistic regression. A stronger microbiological predictor was the positivity of the latest blood culture before operation, which was often a marker of operation being performed before completion of an adequate course of antibiotic therapy, generally because surgery was brought forward for severe cardiac failure or embolic risk. In a post hoc analysis (data not shown), this was significantly associated with positive valve culture, staphylococcal etiology, emergency operation, large vegetations, and persistent signs of infection in the early postoperative period: this latter could be related to undisclosed extracardiac foci and/or particularly high local bacterial load leading to clinically evident blood dissemination following surgical manipulation.

Finally, perivalvular involvement has already been controversially discussed as a possible risk factor for mortality following IE surgery [13, 19]. Indeed, some studies concluded that periannular extension of the infection does not entail a higher risk; however, those were not purely surgical series but also included medically treated patients [19]. Thus, the perivalvular factor might represent not only a marker of more virulent microbial agent but also a predictor of more complex surgery, expectedly implying extensive reconstruction procedures and longer cardio-pulmonary and cross-clamp times, which ultimately increase early mortality risk [20]. As an alternative interpretation, echocardiography can yield a false diagnosis of abscess or even miss its detection, introducing an unquantifiable bias in the analysis of the prognostic value of perivalvular involvement in mixed medical/surgical IE series. Conversely, surgical inspection can provide the definitive confirmation of a true abscess: in the present series, 0.2% of patients had a false preoperative diagnosis of abscess, and a preoperatively unseen abscess was detected in 2% at surgery.

### 4.3. Study Limitations

The main obvious limitation of the present study is the small patient sample. Single-center design ensured uniformity of methods in the perioperative period, which was essential for a study concerning postoperative mortality prediction. However, this limitation restricts the value of the present study to a hypothesis-generating analysis, demonstrating the need to combine more soundly developed predictive systems, like the score introduced by Gaca et al. [11], with more specific subscores including variables unique to IE. Our score may serve to this scope, provided that it will be validated in other larger series, possibly in multicentric design. Since the preoperative features of our patient population, the overall mortality and the predictors emerging from the analysis did not differ importantly from those of many other centers [4, 12–15, 17–20], we believe that our analysis was not affected by significant selection, referral, or treatment biases and that our study population is well representative of the real scenario of native valve IE: this may be a good premise in the perspective of future expansion of the present study in multicentric design. Unfortunately, like many centers in our Country, we have been using the EuroSCORE system, since its introduction, for routine risk prediction in all our surgical practice: this system has been more recently criticized as not adequate to the setting of valve surgery [21]; however a retrospective estimation of the STS score in this series was not possible due to the lack of the relevant data, not prospectively collected. Another limitation (especially to the homogeneity of the population) was the inclusion of a minority of patients with healed endocarditis (17%), namely, patients having discontinued antibiotics after the clinical evidence of no activity of the infection (no fever, negative blood cultures): this small subset was included notwithstanding its expected lower mortality, as a “control” subgroup, to calibrate the reliability of the overall series, as well as to rule out the independent effect of some factors (such as vegetations, for example, which were present at surgery in 46% cases of clinically healed endocarditis) on the study endpoint. Similarly, others have included even much greater proportions (>48%) of healed IE patients in their analysis of mortality predictors [11].

## 5. Conclusions

In the present study we found that IE-specific factors (e.g., cultures, abscess), beside the generic ones (e.g., age, renal impairment, poor hemodynamic conditions), independently predicted mortality following native valve endocarditis surgery. We also ascertained the feasibility of a dedicated stratification tool: our system will be calibrated and validated in multicentric series in the next future. Provided successful validation on a larger scale, adjunctive risk-stratification tools like the model developed in this study could be used along with the EuroSCORE or the STS score, to more specifically define individual IE patient's risk profile.

## Figures and Tables

**Table 1:** Bivariate correlates of hospital (30-day) mortality.

	Deaths (*n*/*N*; %)	*P*
Age: <40 years	2/71 (2.8%)	
40–49 years	6/146 (4.1%)	
50–59 years	7/91 (7.7%)	<0.001
60–69 years	10/81 (12.3%)	
70–79 years	13/45 (28.9%)	
≥80 years	2/6 (33.3%)	

Sex (female)	16/124 (12.9%)	0.10

IE Phase (active)	37/365 (10.1%)	0.09

Site: Aortic	14/200 (7%)	
Mitral	9/110 (8.2%)	
Tricuspid	6/41 (14.6%)	0.27
Mitroaortic	10/71 (14.1%)	
Other	1/18 (5.6%)	

Drug abuse	2/58 (3.4%)	0.07

Diabetes	9/51 (17.6%)	0.03

Preop. renal failure	15/60 (25%)	<0.001

Previous cardiac surgery	4/38 (10.5%)	0.17

Ejection fraction <50%	9/49 (18.4%)	0.03

NYHA class: I, II, or III	14/350 (4.0%)	<0.001
IV	18/70 (25.7%)	

Pre-op. ventilatory support	8/20 (40%)	<0.001

Previous embolism	12/142 (8.5%)	0.86

Cerebral embolism	7/59 (11.9%)	0.46

Emergency operation	17/55 (30.9%)	<0.001

Positive latest preop. blood culture	15/76 (19.7%)	0.001

Isolated microbial agent: Staphylococcal spp.	7/106 (6.6%)	
Streptococcal spp.	8/123 (6.5%)	0.007
Others^1^	12/55 (22%)	

Perivalvular involvement	16/70 (22.9%)	<0.001

Valve repair	2/48 (4.2%)	0.38

Decade: 1980–1990	8/77 (10.4%)	0.33
1990–1999	11/139 (7.9%)	
2000–2009	21/224 (9.4%)	

^1^“Others” here includes gram-negative, corynebacteria, enterococci, fungi, multimicrobial isolates (when introduced in analysis each of these groups constituted a separate modality of the “microbial agent” variable).

**Table 2:** Independent preoperative predictors of mortality (logistic regression analysis) and the deriving scoring system for mortality prediction in native valve IE.

	B coeff.	OR (95% CI)	*P*	Score
Age				
40–49 years				**5 **
50–59 years		1.042 (1.015–1.020)		**7 **
60–69 years	0.041		0.002	**9 **
70–79 years				**11 **
≥80 years				**13**

Renal failure^1^	1.076	3.033 (1.338–6.876)	0.013	**5**

NYHA class IV	1.777	5.913 (2.569–13.612)	<0.001	**9**

Ventilatory support^2^	2.281	9.784 (3.178–30.117)	<0.001	**11**

Positivity of latest pre-op. blood culture^3^	1.093	2.982 (1.304–6.821)	0.010	**5**

Perivalvular involvement^4^	1.110	3.033 (1.338–6.876)	0.008	**5**

^1^Creatinine >2 mg/dL.

^2^Patients admitted to the Cardiac Surgery Department on mechanical ventilation (intubated) or requiring ventilatory support by noninvasive ventilation during preoperative stay (generally for poor hemodynamic conditions and/or pulmonary edema).

^3^This variable identified operation without possibility of previous attainment of negative cultures by antibiotic therapy (latest culture had always been performed within 5 to 7 days preoperatively).

^4^Either annular abscess or aortocavitary fistula.

**Table 3:** Definition of the 4 risk groups according to total score and comparisons in terms of clinically relevant variables.

	Class 1	Class 2	Class 3	Class 4	*P*
Total score range	0–5	7–13^1^	14–19	≥20	

Mean predicted mortality	1 ± 0.7%	3.7 ± 1.6%	12 ± 5%	43 ± 18%	

Age (years)	34 ± 9	55 ± 13	52 ± 17	64 ± 11	<0.001

Sex (female)	30%	20%	37%	39%	0.06
Active IE	81%	79%	91%	89%	0.08

Site: Aortic	47%	44%	51%	41%	0.03
Mitral	27%	27%	16%	22%	
Tricuspid	6%	11%	9%	9%	
Mitroaortic	11%	16%	21%	26%	
Other	9%	2%	3%	2%	

NYHA class IV	—	1.7%	52%	59%	<0.001

Diabetes	3%	12%	22%	18%	<0.001

Renal failure	4%	8%	27%	39%	<0.001

Period: before 2000	39%	40%	14%	7%	0.003
2000–2010	26%	42%	16%	16%	

Positive latest preop. culture	5%	17%	19%	44%	<0.001

Perivalvular involvement	4%	12%	27%	44%	<0.001

Preop. ventilatory support	—	—	6%	30%	<0.001

Emergency	0.7%	4%	22%	59%	<0.001

Infection persistence postop.	2%	2%	9%	29%	<0.001

^1^Note that the 6-point score cannot be obtained by any sum of scores in this system.
